# Analgesic, Anti- inflammatory, Anti- lipoxygenase Activity and Characterization of Three Bioactive Compounds in the Most Active Fraction of *Leptadenia reticulata *(Retz.)Wight & Arn. – A Valuable Medicinal Plant

**Published:** 2015

**Authors:** Sudipta Kumar Mohanty, Mallappa Kumara Swamy, Sushil Kumar Middha, Lokesh Prakash, Balasubramanya Subbanarashiman, Anuradha Maniyam

**Affiliations:** a *Department of Biotechnology, Acharya Nagarjuna University, Nagarjunanagar, Guntur, India.*; b *Padmashree Institute of Management and Sciences, Kommagatta, Bangalore-560060, India.*; c *Rishi Foundation, Jayanagar, Bangalore- 560011, India.*; d *Department of Biotechnology, Maharani Lakshmi Ammanni College for Women, Malleswaram, Bangalore -560012, India.*

**Keywords:** *Leptadenia reticulate*, Anti-inflammatory activity, Analgesic activity, Lipid peroxidation inhibition, Pro-inflammatory cytokines

## Abstract

*Leptadenia reticulata *was reported to be used for several medicinal purposes. The present study was undertaken to evaluate anti-inflammatory, analgesic and lipid peroxidation inhibition activities of *L. reticulata*. The anti-inflammatory assay was performed by λ-carrageenan and formalin induced paw edema test. Pro inflammatory mediators (IL2, IL6, TNF-α) in serum of treated and control organism were analyzed by quantitative ELISA. Lipid peroxidation inhibition was measured by thiobarbituric acid reactive substances (TBARS) assay. Analysis of the most active fraction revealed the presence of one phenolic compound (p-coumaric acid), two flavonoids (rutin and quercetin) which also determined quantitatively. The ethyl acetate fraction at 600 mg/Kg significantly inhibited λ-carrageenan and formalin induced paw edema by 60.59% and 59.24% respectively. Notable reduction in percentage of writhing (76.25%), induced by acetic acid signifies the potent analgesic activity. Lower level of pro-inflammatory cytokines (IL-2, IL-6, TNF-α) in serum at the 4^th^ hour of λ-Carrageenan injection indicated the inhibition of cyclooxigenase-2 (Cox-2), Nitric oxide (NO) and release of prostaglandin to prevent inflammation. The study also demonstrated the decrease in malonaldehyde (MDA) concentration which revealed the lipid peroxidation inhibition potential of the plant. Our finding provides evidence for potent biological activities in tested model which is supported by its characterized bioactive compounds and ethnomedicinal relevance.

## Introduction

*Leptadenia reticulata*, the species used in the current study belongs to Asclepiadaceae family has a unique place due to its multi fold medicinal properties. *Leptadenia reticulata*, is well known for its medicinal value from 4500 to 1600 BC that the oldest scripture in India (Atharva veda) mentioned its utility as a strength giver, maintaining youthful vigour and vitality (1). This plant is reported to be used as one of the ingredient in many herbal formulations (2-4). This valuable medicinal plant has been used for the treatment of various ailments such as hematopoesis, emaciation, dyspnoea, night blindness, fever, burning sensation, inflammation and cancer (1, 5, 6). More than 23 well defined pharmaceutical products and many herbal based formulations are available in the market by using this plant (2, 4). Phyto chemical profile of the plant revealed the presence of several phenolic compounds, glycosides and flavonoids (6, 7). Many bio active compounds such as *α*-amyrin, *β*-amyrin, ferulic acid, diosmetin, rutin, *β*-sitosterol, stigmasterol, hentriacontanol, simiarenol, apigenin, alkaloids, phenolic compounds were isolated from *L. reticulata* (8-10) and reported to have important pharmacological activities. Interest in this plant is further enhanced due to traditional use of this plant for general debility, ulcer, anticancer activities, wound healing activities and anti inflammatory activities (1, 4, 5, 6). However the scientific basis for therapeutic potential and mode of action of the active principles of this species remain still undisclosed. The previous report suggests the presence of phenolic coumpounds and flavonoids in *L. reticulata* (4, 6). The phenolics and flavonoids content of medicinal plants are reported to be responsible for anti inflammatory activities by inducing free radical scavenging activity and reducing inflammatory cytokines (11). In order to validate the therapeutic potential of *Leptadenia reticulata* based on its use in traditional medicine the current research is designed to investigate the anti- inflammatory, analgesic and lipid peroxidation inhibition activity of different solvent extract. HPLC analysis of the most active fraction was carried out to determine the bio active constituents. To our knowledge the present research will be the first report on anti- inflammatory, analgesic and lipid peroxidation inhibition of *L. reticulata *so far.

## Experimental

*Collection of plant material and preparation of extract*

The fresh naturally grown plants of *Leptadenia reticulata *were collected from herbal garden of Padmashree Institute of Management and Sciences, Bangalore. The samples were made in to small pieces and shade dried in controlled condition to avoid other contaminants. The dried samples were ground to fine powder and subjected to aqueous and organic solvent (ethyl acetate, methanol) extraction using soxhlet extractor at temperature not exceeding the boiling point of the respective solvent. The process was continued until complete extraction of active constituents from the powder. The extracts were filtered through Whatman No. 1 filter paper. The filtrate was concentrated using rotary evaporator. The liquid extracts were further evaporated to dryness by vacuum distillation and stored at 4 °C for further use.

*Animals*

Adult Wister Albino rats (200-250 g) of both sexes were chosen for the experiment. The animals were obtained from animal house of Maharani Laxmi Ammani College, Bangalore and all animal experiments were conducted in the research lab of same institute. Animal handling procedures were strictly followed throughout the experiment according to international guidelines. All animals were fasted 24 hours prior of starting the experiments and had free access to water. 

*Acute toxicity study*

The toxic effects of the different extracts of *L. reticulata* were evaluated before further experimentation (12). The ethyl acetate, methanol and aqueous extract were analyzed for acute toxicity profile with reference to any behavioral changes and mortality in Wister albino rats. The protocols were followed according to internationally accepted OECD-423 guidelines. The animals were randomly divided in to six groups with four animals in each. A dose of 1000 mg/Kg and 1500 mg/Kg were administered orally. All animals were fasted 4 hours prior to treatment but had free access to water. After administration of the test drugs, sign of toxicity and mortality was observed with special attention once in a hour for the first four hours and daily four times thereafter for a period of fourteen days. Any symptoms of ill health or mortality were recorded.

*Anti- inflammatory activity*

*λ-Carrageenan induced paw edema test*

Anti-inflamatory activities for different extracts were studied by rat hind paw edema test according to (13). The animals were selected randomly and were divided in to eight groups with four animals in each. Group I serves as control where only vehicle was administered. Group II to IV received (400 mg/Kg) of extract (methanol, ethyl acetate, water) and group V to VII received (600 mg/Kg) of different extract respectively. Group VIII served as positive control by receiving reference drug diclofenac sodium (50 mg/Kg) orally. After one hour of the above treatment paw edema was induced by subplantar injection of 100 µL of λ-Carrageenan (1% carrageenan in 0.9% W/V normal saline) in to the right hind paw of each animal. Paw volumes were measured at 0, 1, 2 and 4 hours by using a plethysmometer (Ugo Basile). The anti- inflammatory potency of different fractions were determined by calculating the edema inhibition percentage with the following equation

$\%\mathrm{inhibition of edema}=\frac{V_{c}-V_{t}}{V_{c}}\times 100$

V_c_ is mean paw volume from control group

V_t_ is mean paw volume from each test group at different time

*Formalin induced paw edema test*

Formalin induced edema test was conducted according to (14). The animals were divided randomly in to groups as carrageenan method. The sample at dose of 400 mg/Kg and 600 mg/Kg or vehicle or standard drug diclofenac sodium (50 mg/Kg) were administered before one hour of formalin injection. 100 µL of formalin (2% in distilled water) was injected in to subplantar region of hind paw of Wister Albino rats. Paw volume of treated animals were measured at 0, 1, 2, 4 hours and compared with control by plethysmographic method. Percentage of edema inhibition was calculated same as carrageenan method.

*Measurement of pro inflammatory mediators*

The serum from each treated animal, control (vehicle received) and positive control (diclorofenac received) were isolated separately at the end of experiment. The level of pro- inflammatory cytokines such as IL2, IL6 and TNF-α were measured in serum by antibody captured ELISA according to kit manufacturer’s instruction (The RayBio ELISA Kit). The antibodies for IL2, IL6 and TNF-α were coated by adding in to 96 well plates and incubated overnight. Next day serum sample followed by secondary antibody and streptavidin- HRP was added to the respective antibody coated well. Wells were washed with buffer in each step to remove the unbound antigen or antibodies. The absorbance for IL2, IL6 at 405 nm and TNF-α at 450 nm were taken in microplate reader. The concentration of IL2, IL6 and TNF-α was determined from the standard curve and expressed as picogram per milligram (pg/mg) of protein.

*Analgesic activity*

*Acetic acid induced writhing test*

The writhing test for analgesic activity was performed according to the method described by (15). The wister albino rats were divided in to eight groups with four randomly selected animals in each group. All animals were fasted 24 h prior to start of the experiment and had free access to water. Group I was treated as control administered with vehicle only, group II was given with standard drug diclofenac sodium (50 mg/Kg) and other groups received extract of ethyl acetate, methanol and water at 400 and 600 mg/Kg subsequently. After one hour of treatment writhing was induced by intraperitoneal injection of 1% acetic acid (1000 µL/Kg body weight). The writhing response was observed by counting the total number of writhes after five minutes of injection of acetic acid during 20 minutes. Data were recorded as mean number of writhes from each group and percentage of inhibition were calculated according to following formula.

$\%\mathrm{inhibition}=\frac{W_{c}-W_{t}}{W_{c}}\times 100$

 W_c_ : Average number of writhing in control group

 W_t_ : Average number of writhing in treated group

*Inhibition of Lipid peroxidation*

The inhibitory effect of different fraction for formation of lipid peroxide was determined using rat liver and brain homogenate by thiobarbituric acid reactive substances (TBARS) assay (16). Wister albino rat was anesthetized and liver and whole brain was dissected. The organs were washed in 1% KCl to remove trace of blood and homogenized in 0.15 M Tris KCl buffer. The homogenate was centrifuged at 8000 rpm for 15 minutes and the supernatant was used for lipid peroxidation assay. The reaction mixture was set up by adding different concentration (200,400,600,800 µg/mL) of plant extract in liver and brain homogenate 0.5 mL (25% W/V), Tris-HCl buffer ( 20 mM, pH 7.0); KCl (30 mM); FeSO_4_(NH_4_)2SO_4_.7H_2_O 0.06 mM) and incubated at 37 °C for half an hour. After incubation period 0.5 mL of SDS (8.1%), 1.5 mL of acetic acid (20% pH 3.5) and 1.5 mL of TBA (0.8%) was added to reaction mixture. The total volume was made up to 5 mL by adding distilled water and incubated in water bath at 95 °C for one hour. After cooling to room temperature 1 mL of distilled water and 5 mL of n- butanol- pyridine mixture (15:1 V/V) was added and shaken vigorously and centrifuged at 3000 rpm for 10 minutes. The butanol- pyridine layer was removed carefully and absorbance was measured at 532 nm. L ascorbic acid was used as positive control. The lipid peroxidation inhibition was calculated by comparing the control absorbance with that of test absorbance. The percentage of inhibition was calculated with according to following formula.

$\%\mathrm{inhibition of edema}=\frac{\mathrm{A control}-\mathrm{A test}}{\mathrm{A control}}\times 100$

A control - absorbance of the control

 A test - absorbance of sample in presence of extract

*HPLC analysis*

Among all the extract tested ethyl acetate extract exhibited highest biological activity therefore only the ethyl acetate fraction was subjected to HPLC analysis for detection of active constituents. The extract was filtered through 0.45 µm syringe filter (Millipore) before injecting in to HPLC system. The HPLC system (Waters, Singapore) consisted of photodiode array detector (W2998), dual pump system (515-waters), temperature control module II (TC2-waters), pump control module (PC2-waters), system controller (EMOAA01712) and a reverse phase HPLC analytical column waters Spherisorb C8 (4.6 X 100 mm) 5 µm particle size. The flow rate was adjusted to 1.0 mL/min, sample run time was 30min and the detector was set at 254nm at 1.2 nm resolution with the mobile phase AcN: H2O: MeOH (70:30:20 v/v, isocratic mode). Standards of p-coumaric acid, rutin and quercetin (25 µg/mL) were injected separately. Active constituents in the samples were identified by comparison of retention time with respective standards. Data was analysed using Empower software (Waters inc.2012).

*Statistical analysis*

All the data were expressed as mean ± standard error. Significant difference between mean were calculated by one way analysis of variance (ANOVA) followed by mean comparison method of Duncan’s multiple range test (DMRT). Data were analyzed by using SAS software version 9.2 (SAS® institute inc., USA). P< 0.05 considered to be statistically significant. 

## Result and Discussion

*Acute toxicity study*

It is imperative to conduct toxicity study for safety and efficacy of herbal product. From the acute toxicity study conducted for ethyl acetate, methanol and aqueous extract of *Leptadenia reticulata*, no mortality or any behavioral changes were observed in tested animals. The result showed that administration of dose level up to 1500 mg/Kg body weight is safe and non toxic. Ethyl alcohol extract of *L. reticulata* at 2000 mg/Kg body weight was reported to be safe (6).

*Anti- inflammatory activity*

*λ – Carrageenan and formalin induced paw edema test*

Though several experimental models are used for anti inflammatory study, we have chosen λ – Carrageenan and formalin induced paw edema model followed by determination of pro inflammatory cytokines (IL-2, IL-6, TNF-α) in serum for detail exploration of the therapeutic potential of *Leptadenia reticulata *as anti- inflammatory drugs. The λ – Carrageenan induced paw edema is most suitable and widely used test for anti-inflammatory activity of natural products (17, 18). The formalin induced paw edema test is another appropriate method for anti- inflammatory and anti arthritic activity which closely resembles to human model (19). 

The mean increase in paw volume and percentage of edema inhibition from 0 h up to 4 hours with different extracts, standard and control is represented (Table 1 and Table 2). The result revealed that extracts tested are having significant (p<0.05) anti-inflammatory activity in rats. Significant increase in percentage of inhibition of edema was observed in the group treated with ethyl acetate fraction followed by methanol. The ethyl acetate extract at test doses 400 mg/Kg and 600 mg/Kg reduced edema by 40.25% and 60.59% respectively. The result of formalin induced paw edema test also proved the higher efficiency of ethyl acetate fraction in suppression of edema with inhibition percentage of 39.91 and 59.24 subsequently. Diclofenac sodiumm (reference drug) exhibited remarkable inhibition percentage of 71.18 and 72.26 in λ- Carrageenan and formalin induced edema test at fourth hour. The Carrageenan induced edema is a biphasic process which is characterized by release of histamine, serotonins in the first phase and prostaglandin, bradykinins, leukitrienes in the second phase (20). The ethyl acetate extract was found to be highly effective. From the above result it is evident that the phytoconstituents responsible for anti-inflammatory activity of this plant are more concentrated in ethyl acetate fraction than other. This also 

correlates our previous studies where the phytochemical screening indicates the presence of phenolics, flavonoids and terpenoids which might add to the biological activity.

**Table 1:** Effect of different extract of *L. reticulata* on λ-Carageenan Induced Paw edema test

**Treatment**	**Increase in paw volume (Time in hours)**				**% Inhibition of Edema**		
(mg/Kg)	0 h	1 h	2 h	4 h	1 h	2 h	4 h
Control	0.65 ± 0.12	1.21 ± 0.76	1.64 ± 0.21	2.38 ± 0.15	-	-	-
EA(400)	0.55 ± 0.34	1.06 ± 0.65	1.11 ± 0.65	1.43 ± 0.43	12 ± 0.12c	32 ± 0.27c	40 ± 0.32c
EA(600)	0.51 ± 0.23	0.89 ± 0.11	0.93 ± 0.77	0.97 ± 0.21	26 ± 0.21b	43 ± 0.31b	59 ± 0.41b
Meth(400)	0.61 ± 0.54	1.16 ± 0.33	1.45 ± 0.19	2.12 ± 0.32	4 ± 0.02d	12 ± 0.12d	11 ± 0.15d
Meth(600)	0.63 ± 0.41	1.13 ± 0.56	1.42 ± 0.16	2.05 ± 0.76	7 ± 0.04d	13 ± 0.11d	14 ± 0.17d
Water(400)	0.62 ± 0.32	1.16 ± 0.85	1.54 ± 0.13	2.14 ± 0.44	4 ± 0.03d	6 ± 0.03e	10 ± 0.12d
Water(600)	0.64 ± 0.22	1.15 ± 0.29	1.57 ± 0.29	2.20 ± 0.55	5 ± 0.06d	4 ± 0.06e	8 ± 0.06e
Diclofenac Sodium (50)	0.46 ± 0.15	0.62 ± 0.19	0.63 ± 0.22	0.66 ± 0.39	49 ± 0.24a	62 ± 0.62a	72 ± 0.65a

F-value 291.50

Note: The Values within each column represent, Mean ± SE followed by same letters in superscript are not significantly different from each other (P< 0.0001). Data analysed by GLM procedure with Duncan’s multiple range test (DMRT) using SAS®.

*** at α = 0.05.

**Table 2:** Effect of different extract of *L. reticulata *on Formalin induced Paw edema test.

**Treatment**	**Increase in paw volume**				**% Inhibition of Edema**		
(mg/Kg)	0 h	1 h	2 h	4 h	1 h	2 h	4 h
Control	0.66±0.87	1.24±0.51	1.62±0.22	2.36±0.88	-	-	-
EA (400)	0.56±0.76	1.02 ±0.49	1.13±0.91	1.41 ±0.72	18±0.11^c^	30±0.2^c^	40±0.21^c^
EA (600)	0.55±0.38	0.82 ±0.97	0.87 ±0.18	0.93±0.17	33.87±0.21^b^	46.29±0.3^b^	60.59±0.43^b^
Meth(400)	0.63±0.51	1.14 ±0.88	1.40±0.19	1.86±0.15	8±0.19^e^	13.58±0.1^d^	21.18±0.19^e^
Meth(600)	0.61±0.44	1.09 ±0.71	1.31 ±0.23	1.58 ±0.63	12.09±0.1^d^	19.13±0.2^d^	33.05±0.23^cd^
Water(400)	0.64±0.57	1.18 ±0.73	1.51 ±0.92	2.15 ±0.75	5±0.12^e^	7±0.18^e^	9±0.16^f^
Water(600)	0.63±0.97	1.17 ±0.61	1.56±0.84	2.19 ±0.95	6±0.1^e^	4±0.15^e^	7±0.13^f^
Diclofenac Sodium (50)	0.49±0.66	0.60±0.11	0.65±0.66	0.68±0.59	52±0.26^a^	60±0.32^a^	71±0.4^a^

F-value 323.85

Note: The Values within each column represent, Mean ± SE followed by same letters in superscript are not significantly different from each other (P< 0.0001). Data analysed by GLM procedure with Duncan’s multiple range test (DMRT) using SAS®.

*** at α = 0.05

*Pro-inflammatory mediators*

 Many medicinal plant and their products have been reported in treatment different acute and chronic inflammatory diseases. Pro inflammatory mediators and cytokines plays crucial role in elicitation of inflammatory reaction. Each of the cytokines (IL2, IL6, TNF-α) tested here are critically involved in controlling local as well as systemic inflammatory response (21). 

**Figure 1:**
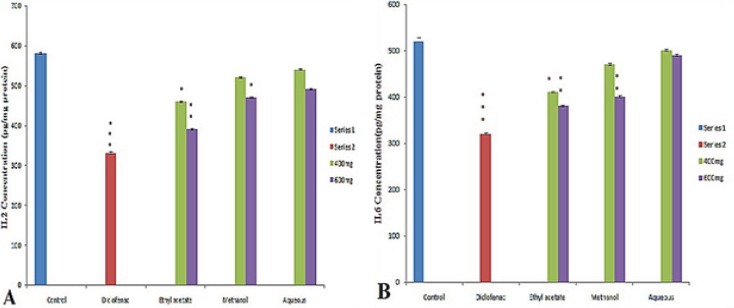
Effect of different extract of *L. reticulata* at 400 mg, 600 mg and Diclofenac on (A) IL2 Concentration (B) IL6 Concentration.

**Figure 2:**
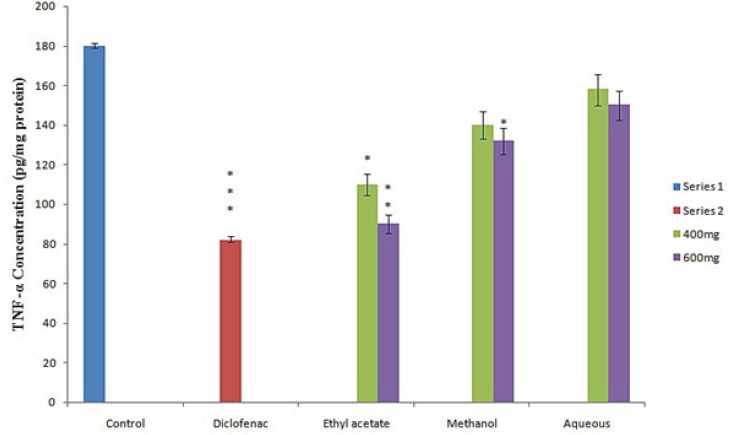
Effect of different extract of *L. reticulata* at 400 mg, 600 mg and Diclofenac on TNF-*α* Concentration.

In the present investigation the regulation of pro-inflammatory cytokines such as IL-2, IL-6 and TNF-*α* in serum were determined to assess the anti-inflammatory activity of different fraction of *Leptadenia reticulata *more clearly. Among the fraction tested the ethyl acetate fraction and reference drug diclofenac sodium results in significant decrease in serum level cytokines (IL2, IL6) (Figure 1A and Figure 1B) and TNF-α (Figure 2). Injection of λ- Carrageenan induces biosynthesis of histamine, serotonin in the first phase (0-1hourr) of edema whereas second phase (1-4 hour) is characterized by secretion of TNF-α, prostaglandin, cox-2 synthesis (22, 23). In the mechanism of inflammation the activation of pro inflammatory enzymes nitric oxide synthase (NOS) and cyclooxigenase-2 (cox-2) by λ-carragennan mainly responsible for elevating prostaglandin level which increase the degree of swelling. It is reported that TNF-α regulates the expression of NOS, cox-2 enzyme and subsequently NO (nitric oxide) and prostaglandins. Thus, evaluation of pro inflammatory mediators provides a good model for screening of anti-inflammatory drugs. The significant decrease in level of pro-inflammatory cytokines (IL2, IL6, TNF- α) in the serum of animals treated with ethyl acetate extract (600 mg/Kg) at 4^th^ hour clearly indicates the anti-inflammatory potential of *L. reticulata*. Some researchers also reported that Carrageenan induced inflammatory response results infiltration of leukocytes such as neutrophils and production of neutrophil derived mediators (24, 25) in the late phase. Thus considering the result from this experiment, the following possible mechanisms are proposed for the anti- inflammatory activity of *L. reticulata*. The primary mechanism of edema inhibition might be due to decrease in the concentration of anti inflammatory mediators such as IL2, IL6 and TNF- α. The reduction in the concentration of TNF- α and IL-6 leads to the inhibition of cox-2, NO and ultimately the release of prostaglandins to prevent inflammation in the second phase. The other possible mechanism could be due to inhibition of free radicals generated like nitric oxide, hydroxyl radicals and superoxide anions which also take part in inducing inflammatory response.

***P<0.001,**P<0.01,*P<0.05 (One way ANOVA and Duncan's multiple range test, DMRT).

***P<0.001,**P<0.01,*P<0.05 (One way ANOVA and Duncan's multiple range test, DMRT).

*Acetic acid induced writhing test*

The ethyl acetate extract of *L. reticulata* (400 µg and 600 µg/mL) exhibited significant (p<0.05) reduction in the number of writhing in a dose dependent manner. The highest inhibition was noted at 600 µg/mL of ethyl acetate extract with 76.25±2.85 percentage of inhibition. The standard drug diclofenac sodium reduced the maximum percentage of writhing with 85.72±2.69 percentage as compared to control (Table 3). The acetic acid induced abdominal constriction is a very sensitive test for analgesic activity of compounds. The mechanism involved here are cyclooxigenase pathway, sympathetic systems (26), peritoneal mast cell (27) and prostaglandin mediated response (28). The highest analgesic activity was achieved by ethyl acetate extract fraction than other fractions. This may be due to the higher concentration of polyphenolics and flavonoids in ethyl acetate fraction. These compounds mediate antioxidant activity by activating antioxidant enzymes like SOD (super oxide dismutase), CAT (catalase), GP (Glutathione peroxidase) and responsible for free radical scavenging activity as free radicals are involved in pain stimulation. It is reported that acetic acid involved in the release of serotonins, bradykinins, prostaglandins and TNF-α which also stimulates pain. In our earlier experiment the successive reduction of TNF-α concentration was noticed which signifies the analgesic activity of *L. reticulata*. 

**Table 3:** Effect of different extract of *L. reticulata* on acetic acid induced writhing test

**Groups**	**Drugs(Dose)** (mg/Kg)	**No .of Wriths (Mean±SE)**	**% Inhibition**
Control	Acetic Acid(1% V/V)	44±6.20^a^	0
Standard	Diclofenac Sodium(50)	6.28±2.40^f^	85.72±2.44^a^
EA	400	18.72±1.46^d^	57.45±1.92^d^
EA	600	10.45±1.23^e^	76.25±1.73^b^
Methanol	400	26.37±2.10^c^	40.06±1.23^e^
Methanol	600	17.63±1.79^d^	59.93±2.12^c^
Water	400	31.27±2.56^b^	28.93±1.46^f^
Water	600	26.44±2.63^c^	39.90±1.48^e^

F-value 338.79

Note: The Values within each column represent, Mean ± SE followed by same letters in superscript are not significantly different from each other (P< 0.0001). Data analysed by GLM procedure with Duncan’s multiple range test (DMRT) using SAS®.

*** at α = 0.05

*Inhibition of lipid peroxidation*

**Figure 3:**
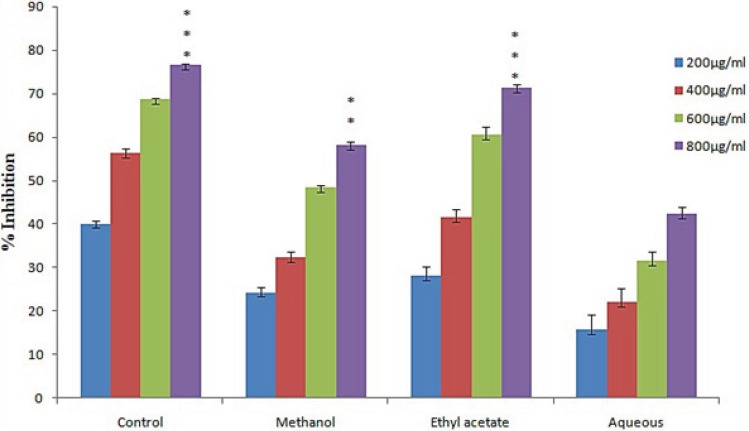
Lipid peroxidation activities of various extract of *L. reticulata*.

It is demonstrated that increase in lipid peroxidation activity trigers damage to brain cells, liver and kidney. The formation of thiobarbituric acid reacting substances (TBRAS) is widely used for mesurment of lipid peroxidation. The malonaldehyde (MDA) formed due to oxidation of polyunsaturated fatty acid reacted with TBA to form pink colour chromogen which is measured at 532 nm. In this study we found that different fraction of *L. reticulata* exhibited lipid peroxidation inhibition activity in a dose dependent manner. The highest inhibition was achieved by ethyl acetate fraction followed by methanol fraction with 71.2% and 58.18% respectively (Figure 3). Remarkable decrease in MDA concentration was noticed in presence of *L. reticulata* extracts. This decrease in concentration of MDA might be due to activation of enzymes involved in antioxidant activity such as CAT, SOD.

*HPLC analysis*

**Figure 4:**
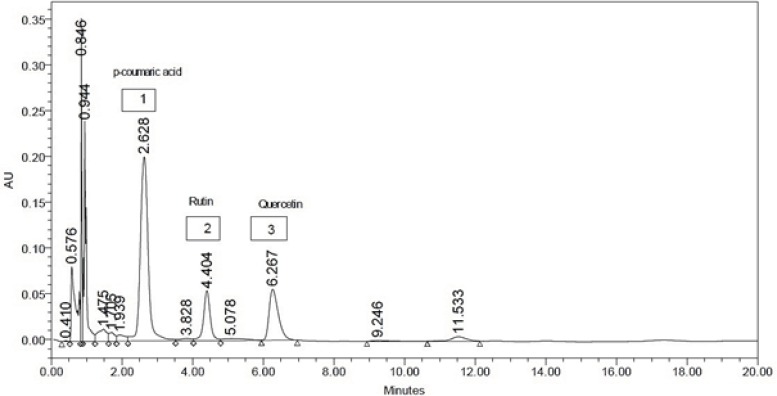
HPLC Chromatogram of Ethyl acetate extract of *L. reticulata*

The ethyl acetate extract exhibited potent biological activity in all the experiments. So the ethyl acetate fraction of *L. reticulata* was subjected to HPLC analysis for detection of important phytochemicals. From HPLC chromatogram (Figure 4) one phenolic compound (p-Coumaric acid) and two flavonoids (rutin, quercetin) were identified with retention time 2.62 minutes, 4.40 minutes and 6.26 minutes respectively. The concentration was calculated from the chromatogram and found to be 24.68 mg (p-Coumaric acid), 12.52 mg (rutin) and 11.84 mg (quercitin) per gram of crude extract. The flavonoids like quercetin and rutin are also reported of having anti- inflammatory, analgesic and anti-oxidant activity (20). The anti-inflammatory effect of flavonoids by inhibiting the enzymes involved in secretion of inflammatory mediators been demonstrated (29). P-Coumaric acid was reported as a unique plant metabolite with remarkable antioxidant and anti-inflammatory properties (30). Only the presences of these compounds in *Leptadenia reticulata* are reported in previous studies (9, 31). In our study we not only reconfirmed the presence of these active phytochemicals but quantitative determination was also made by HPLC method. Thus the presence of phenolic compounds and flavonoids in the fraction of *L. reticulata* might be responsible for anti-inflammatory, analgesic and lipid peroxidation inhibition activities. 

## Conclusion 

The result from the above experiment provides a scientific support of biological activities based on the report of traditional use of *Leptadenia reticulata* for the first time. The result suggests the potential anti-inflammatory, analgesic and lipid peroxidation inhibition activity of this plant. The most possible mechanisms for anti-inflammatory and analgesic activity of the most active fraction (ethyl acetate) were proposed above. The flavonoids (rutin, quercetin) detected by HPLC method are thought to inhibit eicosanoid such as prostaglandin biosynthesis which is the end product of cyclooxigenase path way. The flavonoids also have the ability to inhibit the infiltration of neutrophils and its degranulation there by decreasing the level of arachidonic acid which responsible for painful sensation. Decrease in level of MDA concentration revealed that the extract has ability to increase the activity of antioxidant enzymes such as SOD, CAT, GPx in liver and brain. In conclusion this study supports the mechanism involved in anti-inflammatory and analgesic activity of *Leptadenia reticulata*.
